# Supplementary figures and images for: High Homocysteine Levels Are Associated with Cognitive Impairment in Patients Who Recovered from COVID-19 in the Long Term
Source: J Pers Med. 2023 Mar 10;13(3):503. doi: 10.3390/jpm13030503 (PMC10056581; doi:10.3390/jpm13030503)

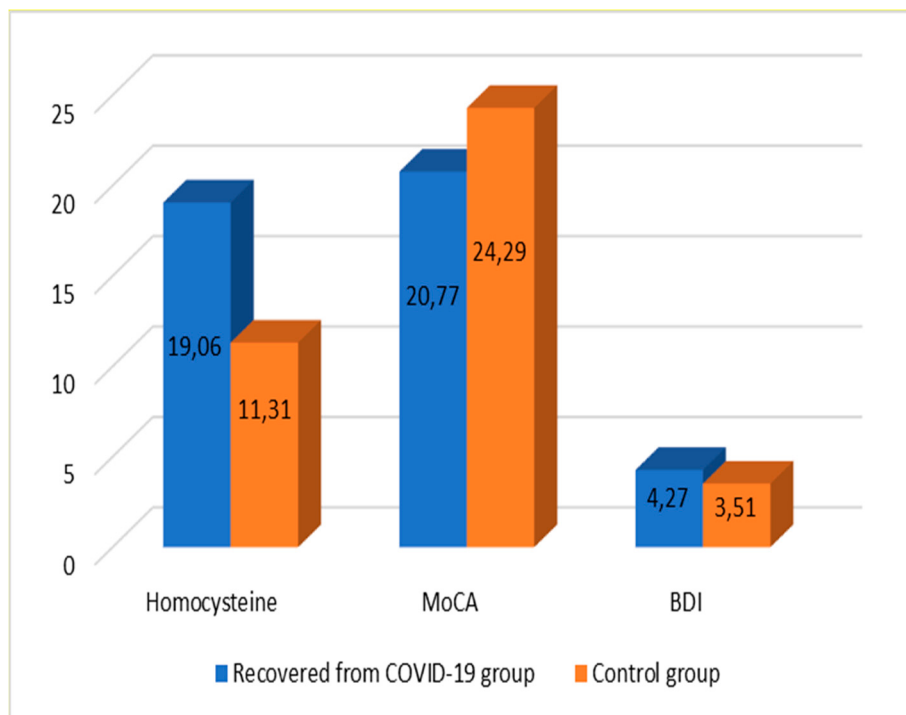

Figure S1: Comparison of homocysteine, MoCA, BDI levels between two groups

Supplement: Supplementary file 1 [file jpm-13-00503-s001.zip › jpm-2242150-supplementary.pdf]
